# Effect of Omega-3 Long Chain Polyunsaturated Fatty Acids (*n*-3 LCPUFA) Supplementation on Cognition in Children and Adolescents: A Systematic Literature Review with a Focus on *n*-3 LCPUFA Blood Values and Dose of DHA and EPA

**DOI:** 10.3390/nu12103115

**Published:** 2020-10-12

**Authors:** Inge S.M. van der Wurff, Barbara J. Meyer, Renate H.M. de Groot

**Affiliations:** 1 Conditions for Lifelong Learning, Faculty of Educational Sciences, Open University of the Netherlands, 6419 Heerlen, The Netherlands; inge.vanderwurff@ou.nl; 2 School of Medicine, Lipid Research Centre, Molecular Horizons, Illawarra Health and Medical Research Institute, University of Wollongong, Wollongong, NSW 2522, Australia; bmeyer@uow.edu.au; 3 NUTRIM School of Nutrition and Translational Research in Metabolism, Maastricht University, 6200 Maastricht, The Netherlands

**Keywords:** adolescents, children, long-chain polyunsaturated fatty acid, cognition, Omega-3 Index

## Abstract

Omega-3 long chain polyunsaturated fatty acids (*n*-3 LCPUFA) supplementation in the cardiovascular field is effective if a certain Omega-3 index (O3I) is achieved or the daily *n*-3 LCPUFA dose is high enough. Whether this applies to studies on cognition in children and adolescents is unclear. The aims of the current review were to investigate whether: (1) a certain O3I level and (2) a minimum daily *n*-3 LCPUFA dose are required to improve cognition in 4–25 year olds. Web of Science and PubMed were searched. Inclusion criteria: placebo controlled randomized controlled trial; participants 4–25 years; supplementation with docosahexaenoic acid (DHA) and/or eicosapentaenoic acid (EPA); assessing cognition; in English and ≥10 participants per treatment arm. Thirty-three studies were included, 21 in typically developing participants, 12 in those with a disorder. A positive effect on cognitive measures was more likely in studies with an increase in O3I to >6%. Half of the studies in typically developing children with daily supplementation dose ≥450 mg DHA + EPA showed improved cognition. For children with a disorder no cut-off value was found. In conclusion, daily supplementation of ≥450 mg DHA + EPA per day and an increase in the O3I to >6% makes it more likely to show efficacy on cognition in children and adolescents.

## 1. Introduction

Numerous studies have investigated the influence of omega-3 long-chain polyunsaturated fatty acids (*n*-3 LCPUFA) on an array of outcomes such as cardiovascular health, depression, attention deficit (hyperactivity) disorder, autism spectrum disorder, pregnancy outcomes, cognition and cognitive development [1,2,3,4,5,6]. The LCPUFA such as docosahexaenoic acid (DHA, 22:6*n*-3) and arachidonic acid (AA, 20:4*n*-6) are important constituents of the brain. Moreover, DHA, AA and eicosapentaenoic acid (EPA, 20:5*n*-3) are involved in bodily and brain processes such as neurite growth, membrane fluidity, blood–brain barrier integrity and inflammation [7,8,9]. The suggested roles of LCPUFA in brain development and functioning have led to a large number of observational studies and supplementation trials in which the relationship between LCPUFA and cognition in children and adolescents was investigated; the results of which were mixed. Some studies show positive effects of supplementation, while others show neutral effects. Most of these studies have been summarized in a multitude of reviews, for example [5,10,11,12,13,14,15,16,17,18,19], yet the conclusions of these reviews are not consistent. Some conclude that there is evidence for an effect of *n*-3 LCPUFA on cognition [5,10,12,14], others conclude that there is only proof for specific subgroups of participants (i.e., infants, elderly, or those with a low *n*-3 LCPUFA status) [16,17,18], and some conclude that there is no effect or results are too mixed to draw conclusions [11,13,15,19]. However, the studies included in these reviews are heterogeneous in their design, various doses of LCPUFA supplements were used, and the composition of the supplements varied greatly. Therefore, it remains uncertain whether *n*-3 LCPUFA supplementation can positively influence cognition and whether the effectiveness could be related to the dose used or the circulating level of *n*-3 LCPUFA in the body, represented by the Omega-3 Index (O3I).

In the area of cardiovascular disease, it has been suggested that recent neutral results in LCPUFA supplementation trials could be due to too low *n*-3 LCPUFA supplementation doses and/or not achieving the cardio-protective O3I range of 8–11% [20]. The O3I is defined as EPA plus DHA in erythrocytes and is based on a standardized analytical method [21]. A target range for the O3I of 8–11% has been suggested as this range is associated with the lowest mortality risk in coronary heart disease [21]. Some have suggested that such a target range could also exist for mental health diseases [22].

In a review of studies investigating the effect of *n*-3 LCPUFA on death from cardiovascular disease, only trials in which the daily dose of DHA was at least 500 mg showed a reduction in cardiovascular mortality [20]. In line with this finding, in the meta-analysis of Emery et al., an effect of *n*-3 LCPUFA supplementation on cognition (i.e., on the domains recall and recognition long-term memory) was only evident when studies with a dose of less than 400 mg DHA + EPA were excluded from the analyses [23]. This indicates that a *n*-3 LCPUFA supplementation dose above 400 mg DHA + EPA might be effective in improving cognition. Nonetheless, Emery et al. were not able to show dose trends for DHA nor EPA for the outcome measures short term memory, attention and inhibition (i.e., a higher dose of EPA or DHA was not related to higher score on short term memory, attention and inhibition tests). However, do note that Emery et al. combined the results of multiple different cognitive tests into one outcome measure. For example, for the outcome measure inhibition results from the KiTap, Stroop test, Matching Familiar Figures tasks, Connors performance test, Test of Variables of Attention, Arrow Flanker Test and Amsterdam Neuropsychological Tasks were combined. Combining such diverse tests can be problematic as even though the tests seem similar, the underlying constructs can be somewhat different and operational differences can also be present [24].

Taking into account the findings with regard to O3I range in cardiovascular health and the limitations of *n*-3 LCPUFA dose in research on cognition, the question was raised whether the mixed results in *n*-3 LCPUFA supplementation studies for cognition could be attributed to the O3I (not reported in the Emery review) or the *n*-3 LCPUFA supplementation dose. Therefore, a systematic literature review was undertaken with the aim (1) to investigate what O3I is needed to improve cognition, and (2) to investigate whether the daily dose of DHA/EPA plays a role in the effect of *n*-3 LCPUFA supplementation on cognition.

The focus of the current review is on *n*-3 LCPUFA supplementation trials in children and adolescents, as the brain develops up to age 25 years and it is to be expected that effects of *n*-3 LCPUFA supplementation on cognition are therefore more pronounced in children and adolescents than in adults [25].

## 2. Materials and Methods

A systematic literature review was conducted. The review protocol was not registered beforehand. Web of Science and PubMed were searched up to the 3th of July 2019 with the following search terms: ‘LCPUFA’, ‘EPA’, ‘DHA’, ‘Omega-3′, in combination with ‘cogniti*, and ‘child*, ‘adolescen*’, ‘toddler’ (for exact search strategy see Appendix A). No date restrictions were applied. Earlier reviews and reviews located in the literature search on LCPUFA and cognition were checked for additional manuscripts to include. The reference lists of included manuscripts were checked for additional manuscripts. Moreover the ‘cited by’ function in Web of Science, or PubMed if the manuscript was not available in Web of Science, was utilized to check whether any of the manuscripts citing the included manuscripts were suitable for inclusion themselves.

Studies were eligible for inclusion if (1) the average age of participants was between 4 and 25 years old; (2) participants (i.e., the child or adolescent) received supplementation with DHA and/or EPA; (3) the study included a measure of cognition; (4) the study was a randomized placebo controlled trial (RCT); (5) cross-over studies had a wash-out period of at least four months as this is the duration which is needed for erythrocyte concentrations to return to pre-supplementation levels [24]; (6) the study included a minimum of 10 participants per treatment arm; (7) the study was published in English.

All manuscripts were scanned by the first author and the following information was extracted: information on study population, supplementation (placebo and active), supplementation duration, adherence percentage, test used to assess cognition, LCPUFA measurement method if included and outcomes.

To compare studies that included blood values, the measured fatty concentrations, if reported, were converted to the O3I with the formulae as suggested by Stark and colleagues [25], called the O3I equivalence from here onwards.

## 3. Results

### 3.1. Included Studies

**Figure 1:**
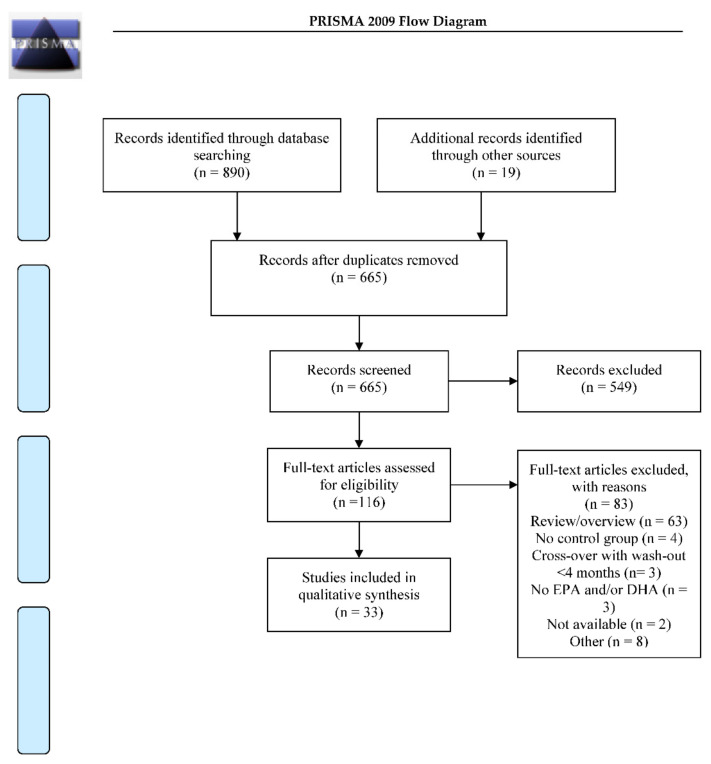
Flow-chart of the search strategy, adapted from [26]. EPA = eicosapentaenoic acid, DHA = docosahexaenoic acid.

The database search yielded 890 manuscripts, and checking of relevant reviews yielded 19 additional manuscripts, after removal of duplicates 665 manuscripts remained (for flow chart see Figure 1). After screening based on title and/or abstract 549 manuscripts were excluded due to not meeting the inclusion criteria. Of 116 manuscripts the whole text was screened, 83 manuscripts were excluded based on the whole text (for reasons see Figure 1). In the end 33 placebo controlled randomized controlled trials (RCTs) investigating the influence of LCPUFA supplementation on cognition were included in the current review. In 21 of these studies, typically developing children and adolescents were included, in 12 the focus was on children and adolescents with a disorder or disease. Of the 12 studies in which the focus was on a disorder or a disease, the vast majority, namely 11 studies, focused on children or adolescents with attention deficit hyperactivity disorder (ADHD). In one study the focus was on children with phenylketonuria (PKU). The majority of studies was either in children (<12 years, *n* = 24) or late adolescents (20–25 years of age, *n* = 8), very few studies focused on early or middle adolescence (12–20 years of age, *n* = 1). The duration of the studies varied from four weeks up to 52 weeks. An overview of the included studies including typically developing participants can be found in Table 1. For an overview of studies including participants with a disorder/disease see Table 2.

### 3.2. Omega-3 Index Equivalence

**Figure 2:**
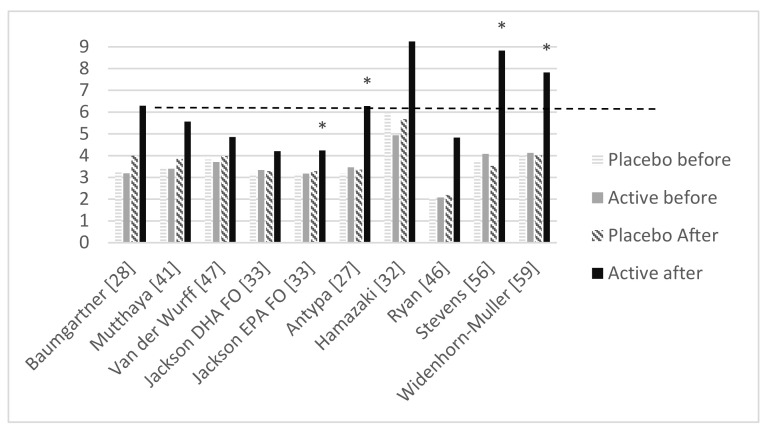
Omega-3 index equivalence at start and end of study for active and placebo group for studies in which blood was collected and recalculation to the Omega-3 index equivalence was possible. Studies in which a positive effect of supplementation was shown are indicated with an *. Note that Jackson et al. are in the figure twice as their study included an eicosapentaenoic acid (EPA) arm and a docosahexaenoic acid (DHA) arm. FO = fish oil.

Of the 33 studies included in this review, 13 studies had no reported blood fatty acid concentrations [29,31,33,35,36,38,43,44,45,49,52,53,55]. In 20 studies, fatty acid concentrations were reported, and the fatty acid concentrations were analyzed in a variety of bodily tissues and blood fractions (note that in some studies multiple fractions were utilized): plasma fatty acids [27,32,33,56], phospholipids in erythrocyte membranes [28,41], plasma phosphatidylcholine [30], erythrocyte phosphatidylcholine [30], erythrocyte phosphatidylethanolamine [30], cheek cells [37,48], capillary whole blood [40,46,47,50], serum phospholipids [42,57,58], total fatty acids in erythrocyte membrane [39,56,59], glycerol-phospholipids in plasma [51] and erythrocytes [54]. Unfortunately, recalculation formulae to the O3I equivalence are only available for a limited number of blood fractions [60]. The recalculation to the O3I equivalence was thus not possible for seven studies because they used a blood fraction for which no recalculation formula is available: erythrocyte and plasma phosphatidylcholine [30], erythrocyte phosphatidylethanolamine [30], cheek cells [37], serum phospholipids [42], cheek cells [48], glycerol-phospholipids in plasma [51] or serum phospholipids [57,58]. Moreover, in three studies only DHA levels were reported and recalculation to the O3I equivalence was thus not possible [39,40,54]. Lastly, one study only reported the baseline fatty acid blood levels [50]. Therefore recalculation to the O3I equivalence was possible for nine out of the 20 studies in which fatty acid values were reported (see Table 3 and Figure 2; Note that Jackson et al. is reported twice in the figure as their study included an EPA-arm and a DHA-arm).

When looking at the nine studies that allowed for recalculation to the O3I equivalence, the increase in the O3I equivalence in the active group varied between 0.87% and 4.75% (see Table 3). Four of the nine studies showed a positive significant effect of supplementation on at least one cognitive measure in the main analyses in the overall sample. 

Figure 2 shows that in three [27,56,59] of those four studies [27,33,56,59] in which a positive effect on cognition was shown, an increase in O3I equivalence was achieved and the O3I equivalence after the intervention was higher than >6% in the active group. There were two studies with an O3I equivalence >6% after supplementation in the active group, however, there were no significant effects of supplementation on cognitive measures [28,32]. The notion that an increase in O3I equivalence is related to improved cognition, is supported by the fact that six [30,37,39,46,57,59] out of the 10 studies [30,37,39,46,47,51,56,57,58,59] that related blood values to cognitive outcomes showed significant positive associations or correlations between (change in) DHA and/or EPA blood values and cognitive tests used.

### 3.3. Supplementation Dose

**Figure 3:**
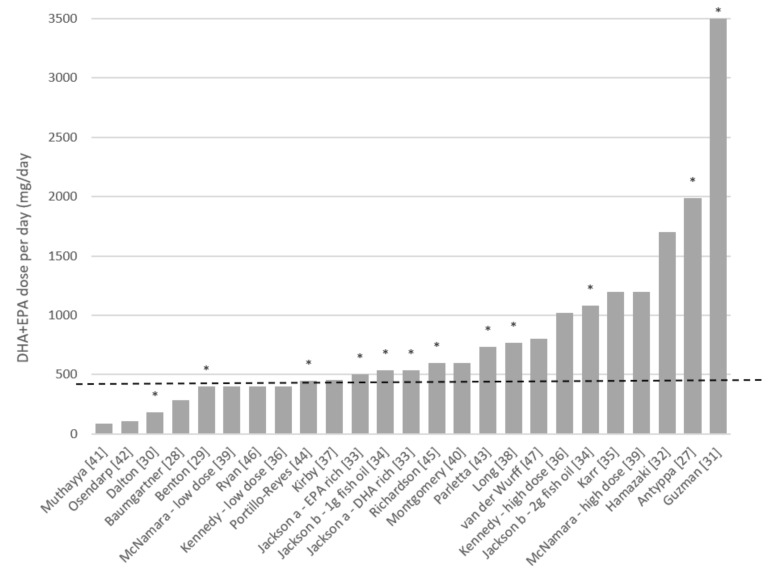
*n*-3 long-chain polyunsaturated fatty acids (LCPUFA) supplementation studies that studied cognition in typically developing children. Studies indicated with a * are those studies in which positive effects of supplementation on cognition are reported. Dotted line indicates supplementation of 450 mg docosahexaenoic acid (DHA) + eicosapentaenoic acid (EPA)per day. Note that in the study of Hamazaki et al. dose differed based on bodyweight. The dose noted here is the lowest dose. Note that Jackson, Kennedy and McNamara included two *n*-3 LCPUFA dose groups, these are reported separately here.

The supplementation dosage of DHA and EPA varied immensely in the 33 included studies, varying from 16.5 mg to 3600 mg DHA per day, and from 0 mg to 1740 mg EPA per day. Supplementation could either contain only DHA or DHA in combination with EPA. Of the 15 studies [27,31,32,33,34,35,36,37,38,39,40,43,44,45,47] in which participants received supplementation of ≥450 mg DHA + EPA per day, 8 (i.e., 53%) showed a positive effect [27,31,33,34,38,43,44,45] (see Figure 3; note that Jackson a and b, Kennedy, and McNamara are represented twice as they included two supplementation groups). In contrast, in only two of the eight studies with a lower supplementation dosage (i.e., <450 mg DHA + EPA per day) a significant effect was shown [29,30]. Moreover, note that in one of those two studies the effect was in favor of the placebo group [29]. While in in six of the eight studies with a daily dosage of <450 mg DHA + EPA per day no significant effects on cognition were shown [28,36,39,41,42,46]. 

### 3.4. Children and Adolescents with a Disease or Disorder

**Figure 4:**
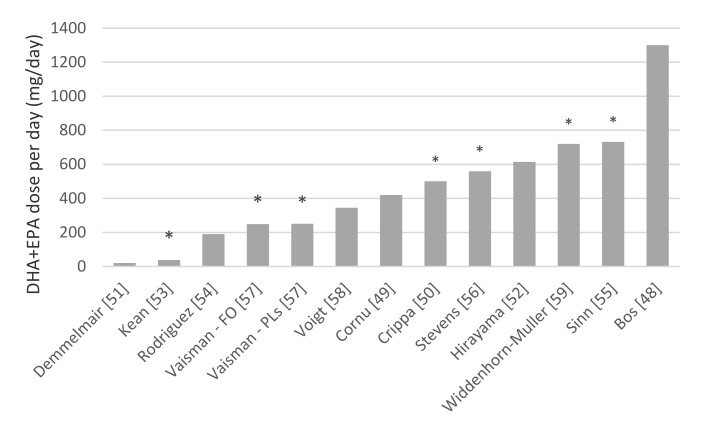
*n*-3 long-chain polyunsaturated fatty acids (LCPUFA) supplementation studies that studied cognition in children and adolescents with a disease or disorder. Studies indicated with a * are those studies in which positive effects of supplementation are reported. Note that in the study of Demmelmair et al. and Cornu et al., dose differed based on bodyweight—the dose noted here is the lowest dose. Note that the study of Vaisman et al. included two *n*-3 LCPUFA dose groups (fish oil (FO) and *n*-3 LCPUFA containing phospholipids (PLs)), these are reported separately here.

Of the 12 studies in which the effect of *n*-3 LCPUFA supplementation on cognition in children and adolescents with a disease or disorder was investigated (see Table 2, note that the study of Vaisman et al. included two *n*-3 LCPUFA dose groups (fish oil (FO) and *n*-3 LCPUFA containing phospholipids (PLs)), these are reported separately in the table), six showed a positive effect on cognition in favor of the *n*-3 LCPUFA supplementation group [50,53,55,56,57,59]. However, in contrast, one study favored the placebo group [52]. In contrast to the studies in which typically developing children and adolescents were investigated, there appeared to be no clear cut-off point above which a positive effect of DHA/EPA supplementation on cognition was shown (see Figure 4). 

## 4. Discussion

The goal of the current review was to investigate what O3I is needed to improve cognition and whether the dose of DHA/EPA plays a role in the effect of *n*-3 LCPUFA supplementation on cognition. There seems to be some evidence that significant cognitive effects of *n*-3 LCPUFA supplementation can only be shown when the post-intervention O3I is increased and reaches at least 6%. However, due to the limited number of studies for which recalculation of the O3I equivalence was possible, this result should be considered with caution. Additionally, there seems to be some evidence that a dosage ≥450 mg DHA + EPA per day does lead to improved cognition in typically developing children and adolescents. For children and adolescents with a disease or disorder, no clear dosage cut-off could be determined.

### 4.1. Omega-3 Index Equivalence

We showed that significant cognitive effects of *n*-3 LCPUFA supplementation can only be shown when the post-intervention O3I is increased and reaches at least 6%. There was only one study in which only a minor increase in O3I equivalence was shown (i.e., the increase in O3I equivalence was 0.87%) and a relatively low O3I equivalence at the end of the intervention for the active group was achieved, but still a positive significant effect on cognition was shown [33]. In this study, both a positive effect of DHA rich supplementation on cognition and a negative effect for both the DHA and EPA rich supplementation was shown. However, in this study, 15 different cognitive tests with a total of 29 outcome measures were reported, without correction for multiple testing. The significant effects could thus reasonably be ascribed to a type I error and should be interpreted with caution. Two studies did have an O3I equivalence >6% after *n*-3 LCPUFA supplementation, but did not show a positive effect on cognitive measures [28,32]. The children in the study of Baumgartner et al. were iron deficient and often undernourished [28], both factors which can negatively influence cognitive performance [61,62,63], and thus mask a potential beneficial effect of the *n*-3 LCPUFA supplementation. The students in the study of Hamazaki et al. had already a rather high O3I equivalence (i.e., O3I equivalence of 5.68% in active group and 5.96% in placebo group) at the start of the study, which makes it less likely to be able to show any effect of supplementation due to a ceiling effect [32]. 

It has also been suggested that an overlap in O3I between placebo and treatment group could cause non-significant treatment effects [64], especially as most authors analyze their results not according to blood values, but according to intention to treat. In this light it is interesting that in the study of Montgomery et al., a significant difference between active and placebo group was shown post intervention for both digit span forward and backward, but change scores in both digit span tests did not differ significantly [40]. It might not be surprising that the change scores did not differ when we consider that there was a clear overlap in DHA blood values between the placebo group and the active group. A similar problem arose in the study of van der Wurff et al., a similar Omega-3 index post-intervention was measured in the placebo and the active group due to non-adherence to the study protocol, which could explain the result of similar cognitive test results in both groups [47].

The fact that an O3I >6% is possibly related to improved cognitive measures is in line with the results found for cardiovascular health and mental health. As mentioned previously, for cardiovascular health, an O3I of >8% was related to the highest cardio-protection, while an O3I < 4% was related to the lowest cardio-protection [21]. Additionally, there are some studies that have shown a positive relationship between increase in *n*-3 LCPUFA blood levels and less depressive symptoms [65,66]. Therefore, it seems likely that to show positive effects of *n*-3 LCPUFA supplementation, a sufficient *n*-3 LCPUFA level in the body should be achieved, which appears to be an O3I of more than 6% for cognition. It is important to note that many of the studies investigating the effect of *n*-3 LCPUFA supplementation on cognition included in this review did not include measurements of *n*-3 LCPUFA levels in the body. Additionally, recalculation formulae to the O3I equivalence are not available for all blood fractions. If future *n*-3 LCPUFA supplementation studies include blood levels, as suggested by the International Society for the Study of Fatty Acids [67], possibly a more precise target range for cognitive functioning can be determined, similar to the one available for cardiovascular health. Additionally, considering the large interpersonal difference in response to LCPUFA supplementation [64,68], it seems prudent that future studies utilize a personalized LCPUFA supplementation dose approach.

### 4.2. Supplementation Dose in Typically Developing Children and Adolescents

The results of the current review point to some evidence that a dose above 450 mg DHA + EPA per day does lead to improved cognition in typically developing children and adolescents. Fifty-three percent of the studies in which children and adolescents received ≥450 mg DHA + EPA showed a positive effect on cognition. However, do note that one of those positive studies was the study of Jackson et al., in which both positive and negative effects of supplementation were shown, also note that these results could be due to a type I error as already mentioned previously [33].

There were six studies with a daily dose ≥450 mg DHA + EPA in which no significant positive effects were shown [32,35,36,37,39,40,47]. One of these studies did show significant effects/associations in secondary analyses, Kirby and colleagues showed significant effects of supplementation on visual attention and impulsivity in those that were compliant with the protocol, while the primary intent to threat analyses did not show significant results [37]. For the other five studies, it is unclear as to why these studies did not show an effect, there was, for example, no clear relationship with supplementation duration or sample size. 

Of the studies with a daily supplementation dose of ≥450 mg DHA + EPA that did show a significant effect, two had a duration of four weeks, one of eight weeks, four of 12 weeks, one of 16 weeks and one of 28.6 weeks. Of the studies with a daily supplementation dose of ≥450 mg DHA + EPA that did not show a significant effect, one had a duration of four weeks, one of eight weeks, one of 12 weeks, two of 16 weeks and one of 52 weeks. Moreover, looking at sample size, no clear relationship could be seen; the sample sizes of the studies ≥450 mg of DHA + EPA that did show a significant effect varied from 34 to 408 participants, while the sample sizes of studies that did not show a significant effect varied from 41 to 376.

One of the two studies that did show a significant effect even though they supplemented with <450 mg DHA + EPA was the study of Dalton et al., in which fish flour was used as supplementation source [30]. Fish flour also includes other nutrients (e.g., protein) which could, especially for the undernourished children included in the study of Dalton et al., provide additional cognitive benefits, which could also explain the significant cognitive findings. The second study with a dose below 450 mg that showed a significant effect was the study of Benton et al. [29]. In this study, it was shown that those taking a DHA supplement for 50 days forgot more words at the end of the study on a word recall test than those that took the placebo. However, this significant effect was not shown in the other memory test which they utilized, the capital recall test. Additionally, from the graphs in the manuscript of Benton et al., it can be deducted that the DHA group already made more mistakes at baseline (it is unclear whether this difference is significant) and that the difference between the DHA and placebo group was rather small (i.e., less than one word). It thus seems unlikely that this indicates an actual negative effect of DHA supplementation on memory performance. 

Note that in the study of Kennedy et al., a significant effect of supplementation was shown for the low dose group of 400 mg DHA + 4 mg EPA per day, but not for the high dose group 1000 mg DHA + 20 mg EPA [36]. After eight weeks of supplementation the children in the low dose group had a significantly higher speed on the Word Recognition test in comparison to the children in the placebo group, while the children in the high dose group had a significantly slower reaction time than the placebo group. For the low dose group this was the case in both the pre- and the post-breakfast session and in the high dose group only in the post-breakfast session. However, as the authors themselves note, the results are inconsistent, it was the only significant effect among 25 cognitive measures (i.e., likely a type I error), the effects were not found on other memory tests, nor on the same task from a different test battery (i.e., the researchers utilized two test batteries). Considering the inconsistent findings and the limitations mentioned above, we classified this study as not-significant. Overall, there is evidence that a dosage above 450 mg DHA + EPA per day does lead to improved cognition in typically developing children and adolescents. 

The finding regarding the positive effects of ≥450 mg DHA + EPA per day for cognition is in line with the earlier review of Emery et al. [23], showing that when studies with a dose of less than 400 mg DHA + EPA were excluded, a small effect size on the domains of recall and recognition long-term memory (i.e., the ability to recognize items after an extended time period) was shown, while this was not the case when studies with a dose <400 mg were included. It is, however, important to note that Emery et al. did not include all studies which were included in this review (i.e., 33 studies were included in the current review of which 16 were also included in Emery et al.). Additionally Emery et al. included studies of children aged 0–4 years, cross-over designs and studies that used foods as form of *n*-3 LPCUFA supplementation (i.e., fish) which were not included in this current review. However, despite the differences in studies included in the review of Emery et al. and this review the conclusion of at least 400 mg and 450 mg of *n*-3 LCPUFA per day are comparable. Furthermore, the current review has additionally shown that an increase in O3 I equivalence and an O3I equivalence >6% makes it more likely to show positive effects of supplementation on cognition in children and adolescents. This finding again points to the importance of including blood values in LCPUFA supplementation trials [67].

The finding from the current study is also in line with the findings for cardiovascular health. Meyer et al. showed that supplementation dosages of >500mg DHA per day lead to cardio-protective effects, but not doses <500 mg per day [20]. The brain can only incorporate about 4 mg DHA per day [69] and only 0.5% of circulating DHA is delivered to the central nervous system [69]. Therefore, to achieve a level of incorporation of 4 mg per day, a relatively high intake of DHA is needed and positive effects of DHA supplementation will only be present with higher doses of supplementation.

### 4.3. Supplementation Dose in Children and Adolescents with a Disease or Disorder

In contrast to the results for typically developing children and adolescents, we were not able to show a clear cut-off for a daily dose of DHA + EPA above which a positive effect on cognition could be shown in children and adolescents with a disease or disorder, although in half of studies (i.e., 6/12 studies) a positive effect was shown. The fact that we could not show a cut-off dose for children with a disease or disorder is surprising, as it has been suggested that *n*-3 LCPUFA supplementation might be more beneficial for those who are underperforming on cognitive and academic measures [45], such as children with ADHD. Note that the vast majority of studies (i.e., 11/12 studies) in children and adolescents with a disease or disorder included in the current review focused on children and adolescents with ADHD. Therefore, this discussion focuses on ADHD. Children with ADHD are known to have impaired executive functions such as inhibition and attention, cognitive functions which are primarily located in the frontal cortex, a brain region especially rich in DHA [70]. However, as ADHD is a multifactorial disorder with a complex etiology, it might be the case that not for all subgroups of children with ADHD a beneficial effect of *n*-3 LCPUFA supplementation could be expected. Lastly, it has been suggested that children with ADHD have lower *n*-3 LCPUFA levels in their body [71] and higher levels of inflammation [72]. It might be that the dose of DHA/EPA utilized in the studies included in this current review were too low. It is important to note that recalculation to the O3I equivalence was only possible for two studies, both of which did show positive effects, and both also had a rather high O3I equivalence in the active group at the end of the intervention (>6%). More research with blood assessments, making recalculation to the O3I equivalence possible, is warranted in children/adolescents with a disease or disorder.

### 4.4. Strengths and Limitations

The strength of this review is the fact that it is the first review in which the relationship between O3I equivalence and cognitive outcomes in children and adolescents is investigated.

However, this review had a number of limitations. Like many previous reviews, we have to conclude that there is large variability in the studies included in this review. The studies differed on various factors: the study population (i.e., age, nutritional status); composition of supplements (i.e., EPA/DHA ratio, but also inclusion of other fatty acids or nutrients); and duration of supplementation. For the assessment of cognition, a wide variety of cognitive tests were used, often with multiple subtests, assessing all different aspects of cognition. Additionally, the number of cognitive measures used varied from one to 25. In the studies with multiple tests, chance findings might be a problem. Moreover, in many studies, blood levels were not assessed or not reported in such a way that recalculation to the O3I equivalence was possible; either a blood fraction for which no recalculation formulae is available was used or the DHA and EPA values were not both reported before and after the intervention. Therefore, only nine out of 33 studies could be compared on the O3I equivalence, which limits the ability to draw conclusions. However, in some studies for which we could not calculate an O3I equivalence, but where blood values were available, the authors executed analyses examining the relationship between blood values and cognitive test outcomes. These analyses relating blood values to cognitive test outcome support our finding: six out of the 10 studies in which those analyses were executed showed significant positive associations or correlations between (change in) DHA and/or EPA blood values and cognitive tests used. Additionally, recently, some controversy regarding the link between the O3I and brain omega-3 level has arisen. In some animal studies, a positive relationships between increases in omega-3 fatty acids in erythrocytes and brain DHA level has been shown [73,74], while in other studies, the correlation between erythrocyte O3I and brain O3I was negative [75]. Whether erythrocyte O3I is related to brain O3I in humans thus remains unsettled. We did not take the source of omega-3 fatty acid supplementation in consideration (i.e., phospholipids, ethyl esters, krill oil, etc.), while some authors have suggested that phospholipids are more efficient in increasing brain levels than other sources of DHA. This could considered to be a limitation, however the notion that different sources of omega-3 fatty acids have different levels of effectiveness in increasing DHA (brain) levels has never been proven conclusively [68]. Due to the large variability in the cognitive tests used, we did not take into consideration what cognitive test was used, which might be considered a limitation of this review. However, combining different cognitive tests into one measure is also not without problems as, even if the tests have a similar underlying construct, the actual construct might be minimally different and operational differences occur, which leads to problems with the comparison of test results [24].

## 5. Conclusions

An increase in the O3I > 6% and daily supplementation of ≥450 mg DHA + EPA makes it more likely to show positive effects of supplementation on cognition in children and adolescents. However, more research is needed where the O3I is measured, to be able to determine a more precise O3I target range for the positive effects on cognitive functioning.

## Figures and Tables

**Table 1:** Overview of studies supplementing children and adolescents with long chain polyunsaturated fatty acids (LCPUFA) and including a cognitive measure as outcome measure.

Author, n ^1^	Mean Age in Years (SD) and Any Specifics	DHA, EPA Dosage Per day Active Group	Other Constituents Active As Specified in Manuscript	Placebo as Specified in Manuscript	Duration (in Weeks)	Cognitive Tests	Adherence	Effect on Cognition?
Antypa n 54 [27]	A: 22.2 P: 22.6	250 mg DHA, 1740 mg EPA,	310 mg other *n*-3 PUFA	Olive oil	4	Affective Go/No-Go task, attentional Go/No-Go task, 15 word test, decision-making (gambling) task.	OS	Y, in gains only trials of gambling task the *n*-3 group showed sig. more risk-seeking decision-making behavior than the placebo group.
Baumgartner n 321 [28]	6–11 iron deficient	240 mg DHA 46 mg EPA ^1,3^	28.6 mg iron sulfate ^2,3^	Medium chain triglycerides ^3^	15	The Atlantis test, Atlantis Delayed test, The Hand Movement test, the Triangles test, The Hopkins Verbal Learning Test	95.4%	N
Benton n 305 [29]	21.8	400 mg DHA	10:0 5 mg, 12:0 38 mg, 14:0 121 mg, 16:0 78 mg, 18:0 10 mg, 18:1 312 mg, 18:2 15 mg, 22:0 3 mg	Maize/soya oil: 16:0 110 mg, 18:0 30 mg, 18:1 233 mg, 18: 2 567 mg, 18:5 41 mg, 20:0 4 mg, 20:1 3 mg, 22:0 2 mg	7.1	Recall of word list (immediate and delayed), recall of capitals, reaction time test, rapid information processing task	NR	Y, those taking DHA sig. forgot more than the placebo group on the recall of word list after 50 days.
Dalton n 183 [30]	7–9 some underweight, wasted or stunted	127.3 mg DHA, 54.6 mg EPA ^4,5^	222.55 mg ALA, 1041.2 mg LA, 15.4 mg AA ^6^	55.8 mg ALA, 9.8 mg EPA, 23.7 mg DHA, 1668.2 mg LA, 4.5 mg AA ^7^	14.9	Hopkins Verbal Learning Test	A: 94.8% P: 94.5%	Y, sig. intervention effect for recall 1 and recall 3, recognition and discrimination index score
Guzman n 34 [31]	23.58 female elite soccer players	3500 g DHA	-	Olive oil	4	Multiple reaction times	NR	Y, those taking DHA showed sig. improvement in complex reaction time and complex reaction efficiency
Hamazaki n 42 [32]	Two groups: (1) 19–20 (2) 22	1500–1800 mg DHA, 200–240 mg EPA dependent on bodyweight	palmitic acid 270–324 mg, oleic acid 219–263 mg, AA 99–119 mg, palmitoleic acid 96–115 mg, stearic acid and others 69–83 mg	Soybean oil + partly deodorized fish oil: LA 1623–1948 mg, oleic acid 669–803 mg, palmitic acid 324–389 mg, ALA 204–245 mg, stearic acid 111–133 mg, DHA and others 15–18 mg	12	Adapted Stroop test, dementia-detecting test (to assess higher functions of brain)	NR	N
Jackson n 140 [33]	DHA rich group 21.96 (0.54), EPA rich group 22.74 (0.61) P: 21.94 (0.50)	I: DHA rich group: 450 mg DHA, 90 mg EPA II: EPA rich group: 300 mg EPA, 200 mg DHA	I: NS II: NS	1 g olive oil	12	Immediate Word Recall, Simple RT, Choice RT, Four Choice RT, Stroop Task, Verbal Fluency, Alphabetic Working Memory, Corsi Block Task, Three Block Task, Telephone Number Working Memory Task, Delay Word Recall, Delayed Word Recognition, Delayed Picture Recognition, Names-To-Faces Recall, Cognitive Demand Battery (Serial 3 Subtractions, Serial 7 Subtractions, Rapid Visual Information Processing).	DHA: 91% EPA: 90% P: 92%	Y, DHA group had sig faster reaction time on Stroop Task compared to placebo group. Both DHA and EPA group matched sig. fewer items on the Names-To-Faces-task than the placebo group.
Jackson n 64 [34]	20.58	1 g fish oil (FO) group: 450 mg DHA, 90 mg EPA, oil. 2 g FO group: 900 mg DHA, 180 mg EPA,	1 g FO: 1 g olive 2 g FO: NS	2 g olive oil	12	Corsi blocks task, Numeric Working Memory, 3-Back Task, Simple Reaction time, Choice Reaction Time, Four Choice Reaction Time, Stroop Task, Rapid Visual Information Processing Task, Serial 7 Subtractions while near-infra red spectroscopy was executed.	1 g: 91% 2 g: 92% P: 96%	Y, sig. treatment effect on reaction time for Choice Reaction Time task, reaction time in both treatment groups faster. Sig. effect of treatment on Rapid Visual Information Processing Task, reaction time sig faster in 2 g FO group compared to placebo.
Karr n 41 [35]	FO: 19.9 (1.8) P: 20.4 (1.6)	480 mg DHA, 720 mg EPA	NS	1 g coconut oil	4	Rey Auditory Verbal Learning Test, Stroop Color and Word Test and Trail Making Test	99%	Y, interaction condition x test moment sign. for Rey auditory Verbal Learning Test stage 6, stage 7 and summary 7–5 (FO improved from baseline, placebo not) but main effect cognition not sign. Placebo condition improved sig. over FO group on Trial Making Test.
Kennedy n 90 [36]	10–12	Low dose: 400 mg DHA, 8 mg EPA High dose: 1000 mg DHA, 20 mg EPA	Low dose: 2092 mg vegetable oil High dose: 1480 mg vegetable oil ^8^	2.5 g vegetable oil containing 75 mg ALA and 1250 mg LA	8	Internet Battery (Word Presentation, Picture Presentation, Arrow Reaction Time Test, Arrow Flanker Test, Paired Associate Learning, Sentence Verification, Delayed Word Recognition, Delayed Picture Recognition) and Cognitive Drug Research Battery (Picture Presentation, Word Presentation/Immediate Word Recall, Simple RT, Digit Vigilance Task: Choice RT, Spatial Working Memory, Numeric Working Memory, Delayed Word Recall, Delayed Word Recognition, Delayed Picture Recognition	>80%	N, sig. main treatment effect on speed of Word Recognition of the Cognitive Drug Research Battery. Post-hoc analysis: the low dose group was sig. faster than the placebo group. However, the high dose group was sig. slower than placebo group.
Kirby n 348 [37]	8–10	400 mg DHA, 56 mg EPA,	64 mg other *n*-3 fatty acids, vitamin A 800 mg RE, vitamin C 60 mg, vitamin D 5 mg, and vitamin E 3 mg	Olive oil	16	Working Memory Test Battery for Children (digit recall, block recall and backward digit recall), TEA-Ch (Creature Counting), Matching Familiar Figures Task	OS	Y, in intention to treat analysis no sig. effects. In per protocol analysis PUFA group had higher number of first correct responses on matching familiar figures task in comparison to baseline, the change for placebo group was not sig.
Long n 202 [38]	20.9	672 mg DHA, 93.3 mg EPA ^9^	14:0 4.5 mg, 16:0 13.8 mg, 16:1 5.4 mg, 17:0 3 mg, 18:0 39.3 mg, 18:1 70.2 mg, 18:0 12.6 mg, 18:2 10.2 mg, 18:3 4.2 mg, 18:4 5.1 mg, 20:0 8.4 mg, 20:1 34.8 mg, 20:2 (*n*-9) 5.4 mg, 20:3 (*n*-6) 2.7 mg, 20:4 (*n*-6) 27.9 mg, 20:3 (*n*-3) 3.3 mg, 20:4 (*n*-3) 8.4 mg, 22:0 (*n*-3) 5.1 mg, 22:1 (*n*-11) 27.9 mg, 22:1 (*n*-9) 6.3 mg, 22:4 (*n*-6) 6 mg, 22:5 (*n*-6) 50.7 mg, 22:5 (*n*-3) 35.7 mg, 24:0 4.5 mg, 24:1 20.4 mg, minor components 4.9%, alpha tocopheryl acetate (2.25 mg), and mixed tocopherols (4.5 mg). multi vitamin and mineral supplement	14:0 9.0 mg, 16:0 392.1 mg, 16:1 2.1 mg, 18:0 50.1 mg, 18:1 562.5 mg, 18:1 (*n*-7) 16.5 mg, 18:2 289.5 mg, 18:3 29.1 mg, 20:0 5.4 mg, 20:1 5.4 mg, 22:0 (*n*-3) 3.3 mg, 22:1 1.5 mg, 24:0 1.5 mg, minor components (0.4%), alpha tocopheryl acetate (2.28 mg), and mixed tocopherols (4.5 mg)	12	Go Stop Impulsivity Paradigm	NR	Y, only for participants with high impulsivity at baseline: the % of inhibited responses was sig. greater if they had consumed DHA rather than a placebo. For participants with low impulsivity no effect of supplementation was shown.
McNamara n 38 [39]	8–10	Low dose: 400 mg DHA High dose: 1200 mg DHA,	Low dose: 2 g corn oil High dose: -	3 g corn oil	8	The identical-pairs version of the Continuous Performance task (CPT-IP).	NR	N
Montgomery n 376 [40]	7–9 underperforming on reading	600 mg DHA 22.5 mg EPA	14:0 72.4 mg, 16:0 222 mg, 18:0 14.5 mg, 18:1 *n*-9 242.9 mg, 18:2 *n*-6 16.1 mg, 22:5 *n*-6 252.5 mg	1500 mg corn/soy bean oil	16	Recall of digits Forward and Recall of Digits Backwards	75%	N
Muthayya n 598 [41]	6–10 marginally nourished	High *n*-3: 86 mg DHA ^10,11^	High *n*-3: 800 mg ALA + high or low micronutrient ^12,13^	Low *n*-3: 120 mg ALA + high or low micronutrient ^13,14^	52	Kaufman Assessment Battery for Children (pattern reasoning, triangles, rover, number recall, word order and Atlantis), Wechsler Intelligence Scales for Children (picture arrangement and coding), Rey Auditory Verbal Learning test (auditory-verbal learning test), Neuropsychological Assessment Tool (verbal fluency) and number cancellation	70%	N
Osendarp n 780 [42]	6–10 Some marginally nourished	88 mg DHA 22 mg EPA ^15,16^	Multivitamin ^17^	Base powder	52	Digits Backwards, Visual Attention 2, Coding, Block Design, Fluency Structured and Random, Rey Auditory Verbal Learning Test.	73–87%	N
Parletta n 408 [43]	3–13 ^18^	174 mg DHA 558 mg EPA	60 mg GLA, 10.8 mg vitamin E	Palm oil + fraction of fish oil	28.6	Draw-A-Person		Y, sig. treatment by group interaction whereby the treatment group improved compared to placebo.
Portillo-Reyes n 55 [44]	8–12 Malnourished	180 mg DHA 270 mg EPA	Not reported	Soy bean oil	12	Symbol Search, Embedded Figures Test, Visual Closure, Block Design, TMT A, Letter Cancellation, Rey Complex Figure, Word List, Semantic Fluency, Matrix Reasoning, Letter-Number Sequencing, Stroop Color and Word test, TMT-B: Shifting.	NR	Y, only the treatment group improved sig. in Symbol Search, Embedded Figures, Visual Closure, Block Design, Stroop-Color, Stroop Color-Word and Matrix Reasoning.
Richardson n 362 [45]	7–9 underperforming on reading	600 mg DHA	NS	1500 mg corn/soy bean oil	16	Recall of Digits Forward and Recall of Digits Backwards	75%	Y, post intervention score for Recall of Digits Forwards sig. higher in active treatment group.
Ryan n 202 [46]	4	400 mg DHA		high-oleic sunflower oil	16	Leiter-R Test of Sustained Attention, Day-Night Stroop Test, and Conners’ Kiddie Continuous Performance Test, Peabody Picture Vocabulary Test.	Nearly 100%	N
Van der Wurff n 267 [47]	13–15	280 mg DHA, 520 mg EPA	NS	Capsules with a fatty acid profile comparable to the fatty acid composition of the average European diet: mix of olive oil, corn oil, palm oil and medium chain triglycerides: (16:0 26%, 18:0 4.6%, 18: 1–9 35.8%, 18:2–6 16.7%, 18:3–32.1%, 20:4–6 0% and other compounds 14.8%)	52	Letter Digit Substitution Task, Concept Shifting Task, Digit Span Forwards and Backwards, D2 test of Attention.	OS	N

A= active, AA = arachidonic acid ALA = α-Linolenic acid, DHA = docosahexaenoic acid, EPA = eicosapentaenoic acid, GLA = Gamma-linolenic acid, HVLT = Hopkins Verbal Learning test, LA = linoleic acid, MFFT = Matching Familiar Figures Task, *n*-3 PUFA = *n*-3 polyunsaturated fatty acids, N = no, NR, not reported, NS= not specified, OS = otherwise specified, P = placebo, RT = reaction time, TMT-B = Trail Making Task part b, Y = yes. ^●^ Note that in some studies also other measures were included such as academic achievement tests (spelling, reading, math), mood measures and more, these are not reported here. ^1^ Recalculated, participants received 420 mg DHA and 80 mg EPA per day four days a week, ^2^ Recalculated participants received 50 mg per day four days a week ^3^ The study was a 2 × 2 factorial design, participants received DHA + EPA+ iron, or DHA + EPA + placebo, or placebo + iron, or placebo + placebo, ^4^ The children received the supplementation in the form of buttery spread which was spread over two slices of bread. ^5^Recalculated, participants received 191.7 mg DHA and 82.2 mg EPA per day approximately 4.65 days per week, 104days in a 6 months period. ^6^Recalculated participants received 335 mg ALA, 1567.36 mg LA and 23.25 mg AA per day approximately 4.65 days per week, 104 days in a 6 months period. ^7^ Recalculated participants received 84 mg ALA, 14.8 mg EPA, 35.6 mg DHA, 2511.3 mg LA and 6.8 mg AA per day approximately 4.65 days per week, 104 days in a 6 months period. ^8^ The amount of LA and ALA in the placebo vegetable oil was specified, this was not the case for the vegetable oil in the active supplement, the low dose group consumed 45 mg ALA and 750 mg LA from the placebo capsules they consumed. ^9^ The study was a 2 × 2 factorial design, participants received vitamins/minerals + DHA, DHA + placebo, vitamins/minerals + placebo, or placebo + placebo; the multi vitamin mineral supplement contained vitamins A (800 mg), B1 (1.4 mg), B2 (1.75 mg), B6 (2 mg), B12 (2.5 mg), biotin (62.5 mg), folic acid (200 mg), niacin (20 mg), C (100 mg), D (5 mg), E (15 mg), K (30 mg), pantothenic acid (7.5 mg), calcium (162 mg), phosphorus (125 mg), magnesium (100 mg), potassium (40 mg), chloride (36.3 mg). iron (5 mg), iodine (100 mg), copper (500 mg), manganese (2 mg), chromium (40 mg), molybdenum (50 mg), selenium (30 mg), zinc (5 mg) and lutein (500 mg). ^10^ The study was a 2 × 2 factorial design, participants received high micronutrient + high *n*-3 fatty acid treatment, low micronutrient + high *n*-3 fatty acid treatment, high micronutrient + low *n*-3 fatty acid treatment or low micronutrient + low *n*-3 fatty acid treatment. All participants additionally received a fruit flavoured biscuit with a creamy filling and a flavoured milk powder drink providing 420 kcal and 13.5 g protein 9.3 g fat (3.7 g *n*-6 fatty acids), and 3.6 mg vitamin E per day. ^11^ Recalculated, participants received 100 mg DHA six days a week. ^12^ Recalculated participants received 930 mg ALA six days a week. ^13^ High micronutrient per day: vitamin A 428.6 µg RE, riboflavin 0.77 mg, vitamin B6 0.86 mg, vitamin B12 1.54 µg, folate 257.1 µg, vitamin C 194.7 mg, calcium 198 mg, iodine 85.7 µg, iron 15.4 mg, zinc 9 mg. Low micronutrient per day: vitamin A 64.3µg RE, riboflavin 0.12 mg, vitamin B6 0.13 mg, vitamin B12 0.23 µg, folate 38.6µg, vitamin C 4.5 mg, calcium 90 mg, iodine 12.9 µg, iron 2.3 mg, zinc 1.5 mg. ^14^ Recalculated participants received 140 mg ALA per day, six days a week. ^15^ The study was a 2 × 2 factorial design, participants received mix of micronutrients, *n*-3 fatty acids, both or none; the intervention product were added to a base powder that contained 8 g of protein, 12 g sugar and 4 g maltodextrin, additionally in Indonesia participants received three biscuit providing 100 kcal. ^16^ Children in Indonesia received the intervention product six days a week, recalculated per day: 75.4 mg DHA, 18.86 mg EPA. ^17^ Multivitamin contained: Iron 10 mg, Zinc sulfate 5 mg, vitamin A as retinol acetate 400µg RE, folate 150 µg, vitamin B6 1 mg, vitamin B12 1.5 µg, vitamin C 45 mg, children in Indonesia received the intervention product six days a week, recalculated per day: Iron 8.6 mg, Zinc sulfate 4.3 mg, vitamin A as retinol acetate 342.9 µg RE, folate 128.6 µg, vitamin B6 0.86 mg, vitamin B12 1.29 µg, vitamin C 38.6 mg. ^18^ Only two out of 408 participants were three years old, it was therefore decided to include this study despite inclusion criteria.

**Table 2:** Overview of studies supplementing children and adolescents with a disease or disorder with LCPUFA and including a cognitive measure as outcome measure.

Author, n, Reference	Mean Age in Years (Sd) and Any Specifics	DHA, EPA Dosage per Day Active Group	Other Constituents Active as Specified in Manuscript	Placebo as Specified in Manuscript	Duration (in Weeks)	Cognitive Measure	Adherence	Effect on Cognition?
Bos n 79 [48]	ADHD: 10.3 Typically developing: 10.9	650 mg DHA, 650 mg EPA	70 mg ALA, 30 mg AA, 1 g LA, 2.66 g saturated FA, 2.08 g MUFA, 6.4 mg vitamin E	60 mg ALA, 1 g LA, 2.59 g saturated FA, 4.32 g MUFA, 0.52 mg vitamin E	16	Go-No Go task	ADHD: 92.2% TD: 91.4%	N
Cornu n 162 [49]	A: 10.2 P: 9.7 ADHD	84 mg-168 mg DHA, 336–672 mg EPA^1^	100μg vitamin A, 1.25 μg vitamin D, 3.5 mg vitamin E	Olive oil + marine lipid concentrate containing 4.83 mg EPA + DHA, 100μg vitamin A, 1.25μg vitamin D, 3.5 mg vitamin E	12	KiTAP (6–10 yr) and TAP (11–15 yr): Distractibility, Flexibility and Go/NoGo	OS	N
Crippa n 50 [50]	7–14 ADHD drug-naïve	500 mg DHA		wheat germ oil, low concentration vitamin E.	24	Abbreviated ANT: Focused Attention 4 letters, Shifting Attentional Set-Visual and Sustained Attention.	83.2%	Y, DHA group showed a decrease in misses in Focused Attention 4 letters task at 6 mo, placebo group showed decrease at 4 mo but not at 6 mo. DHA group showed reduction in false alarms irrelevant target in Focused Attention 4-letters task at 6 mo.
Demmelmair n 109 [51]	5–13 PKU	Between 20 and 254 mg DHA^2^	14:0 5–64 mg, 14:1 0–1 mg, 16:0 37–61 mg, 16:1 1.5–14 mg 18:0 15.5–20 mg, 18:1 403–536 mg, 18:2 *n*-6 29–35, 18:n3–3 0.5–1 mg, 20:0 1.5–2 mg, 20:1 *n*-9 1–2 mg, 22:0 3.5–5 mg, 24:1 *n*-9 1–2 mg, 22:5 *n*-3 0–3 mg^3^	16:0 16.5–33 mg, 16.1 0.5–1 mg, 18:0 16–32 mg, 18:1 428.5–857 mg, 18:2 *n*-6 29–58 mg, 18:3 *n*-3 0.5–1 mg, 20:0 1.5–3 mg, 20:1 *n*-9 1.5–3 mg, 22:0 4–8 mg, 24:1 *n*-9 1.5–3 mg^4^	24	Raven Progressive Matrices (standard or coloured)	96–102%	N
Hirayama n 40 [52]	6–12 ADHD	514 mg DHA, 100 mg EPA^5^	-	Olive oil	16	Adapted Test of Visual Perception, Visual and Auditory Short-Term Memory, Developmental Test of Visual–Motor Integration, Continuous Performance Test	NR	Y, Visual Short Term Memory and number of errors of commission on Continuous Performance Test improved sig. in control group.
Kean n 112 [53]	6–14, mean 8.7 ADHD rating score >15	16.5–22 mg DHA, 21.9–29.2 mg EPA ^6^	300–400 mg olive oil, 0.68–0.90 mg vitamin E, diverse sterol esters (amounts not specified)	Olive oil, lecithin, coconut oil and beta-carotene	14	COMPASS cognitive battery: Word Presentation, Immediate Word Recall, Picture Presentation, Simple Reaction Time, Choice Reaction Time, Numeric Working Memory, Delayed Word Recall, Delayed Word Recognition, Delayed Picture Recognition. TOVA	96.7%	Y, improved memory accuracy score in active group for recalled target and non-target pictures correctly and sig. overall picture recognition accuracy only at 8 weeks not at 14 weeks. No sig. differences in TOVA. Additionally subgroup differences showed difference between diagnosed and non-diagnosed participants in response to supplement.
Rodriguez n 95 [54]	6–19 ADHD	1000–2000 mg DHA, 90–180 mg EPA ^7^	150–300 mg *n*-3 DPA, 4.5–9 mg vitamin E	Olive oil	24	AULA Nesplora test, d2 test of attention	OS	N
Sinn n 167 [55]	7–12 ADHD symptoms no stimulant medication	174 mg DHA, 558 mg EPA ^8^	60 mg GLA, 10.8 mg vitamin E, multivitamin ^9^	Palm oil: 44.3% palmitic acid C16, 4.6% stearic acid C18, 1% myristic acid C14, 38.7% oleic acid C18, and 10.5% LA	15w–one way CO 15w	Digit-Symbol Coding, Inspection Time, Rey Auditory-Verbal Learning Test, Creature Counting, DSB, Knock and Tap, Stroop Colour-Word Test.	OS	Y, PUFA group had sig improvement compared to placebo on Creature Counting with large effect.
Stevens n 50 [56]	P: 10.1 A: 9.5 ADHD + high thirst/ skin symptom score	480 mg DHA, 80 mg EPA	40 mg AA, 96 mg GLA, 24 mg vitamin E	Olive oil	16	Conners’ Continuous Performance Test, Woodcock-Johnson Psycho-Educational Battery-Revised	88%	Y, hit reaction time on Conner’s Continuous Performance Test showed a treatment effect in favor of treatment group. Auditory processing showed improvement in treatment group only (no significant difference between groups).
Vaisman n 83 [57]	8–13 ADHD and impaired visual sustained attention performance	FO: 96 mg DHA, 153 mg EPA or *n*-3 LCPUFA containing PLs: 95 mg DHA, 156 mg EPA	FO: 14:0 63 mg, 16:0 147 mg, 18:0 31 mg, 20:0 2 mg, 22:0 2 mg, 24:0 4 mg, 16:1 *n*-7 68 mg, 18:1 *n*-9 95 mg, 18:1 *n*-7 25 mg, 20:1 *n*-9 12 mg, 22:1 *n*-9 7 mg, 18:2 *n*-6 18 mg, 18:3 *n*-6 2 mg, 20:4 *n*-6 7 mg, 18:3 *n*-3 25 mg LC-PUFA containing PLs: 14:0 19 mg, 16:0 141 mg, 18:0 7 mg, 22:0 1 mg, 16:1 *n*-7 19 mg, 18:1 *n*-9 37 mg, 18:1 *n*-7 44 mg, 20:1 *n*-9 5 mg, 22:1 *n*-9 5 mg, 18:2 *n*-6 11 mg, 18:3 *n*-6 1 mg, 20:4 *n*-6 4 mg, 18:3 *n*-3 7 mg, 22:5 *n*-3: 4 mg ^10^	16:0 30 mg, 18:0 13 mg, 20:0 5 mg, 22:0 3 mg, 24:0 1 mg, 16:1 *n*-7 1 mg, 18:1 *n*-9 415 mg, 20:1 *n*-9 12 mg, 22:1 *n*-9 4 mg, 18:2 *n*-6 150 mg, 18:3 *n*-3 69 mg	12	TOVA	OS	Y, The PL-n3 and, to a limited extent, the FO treatment significantly improved errors of commission, response time, and response time variability as compared with placebo intervention
Voigt n 63 [58]	6–12 ADHD	345 mg DHA	-	Not specified	16	TOVA, The Children’s Color Trails test	OS	N
Widdenhorn-Muller n 110 [59]	6–12 ADHD	120 mg DHA, 600 mg EPA	15 mg vitamin E	Olive oil	16	DSF, DSB, Letter Number Sequencing, HAWIK Number-Symbol, Symbol Search Test, KITAP or TAP (dependent on age participant)	OS	Y, sig. improvement on working memory index score, Digit Span subtest and DSB.

A = active, AA = arachidonic acid, ADHD = attention deficit hyperactivity disorder, CO = cross over, COMPASS = computerized mental performance assessment system, DHA = docosahexaenoic acid, DSB = digit span backwards, DSF = digit span forwards, EPA = eicosapentaenoic acid, FA = fatty acids, FO = fish oil, HVLT = Hopkins Verbal Learning test, KITAP = “TestBatterie zur Aufmerksamkeitsprüfung für Kinder”, LCPUFA = long-chain polyunsaturated fatty acids, MVM = multivitamins/minerals, N = no, NR = not reported, OS = otherwise specified, P = placebo, PKU = phenylketonuria, PLs = phospholipids, RT = reaction time, MFFT = Matching Familiar Figures Task, TAP = “Testbatterie zur Aufmerksamkeitsprüfung”, MUFA = monounsaturated fatty acids, TOVA = Test of Variables of Attention, TD = typically developing, Y = yes. ^●^ Note that in some studies also other measures were included such as academic achievement tests (spelling, reading, math), mood measures and more, these are not reported here ^1^ Dosages depended on age 6–8 yr 336 mg EPA and 84 mg DHA, 9–11 yr 504 mg EPA, 126 mg DHA, 12–15 yr 672 mg EPA, 168 mg DHA. ^2^ There were five different capsules (0, 20, b3,80 or 127 mg DHA per capsule) and participants <35 kg consumed one capsule per day, those >35 kg consumed two capsules per day. ^3^ The values reported here are the amount of fatty acids for the lowest DHA dose and the highest DHA dose. ^4^ Values reported are for the low and high placebo dose. ^5^ Recalculated 3600 mg DHA, 700 mg EPA per week ^6^ Participants <45 kg received three capsules per day, participants >45 kg received four capsules per day, note that PCSO-524 extract of the New-Zealand green-lipped mussel was used. ^7^ Participants <32 kg received one sachet per day, participants >32 kg received two sachets per day. ^8^ There were three study groups: PUFA, PUFA + multivitamins/minerals and placebo. ^9^ Multivitamin/mineral supplement contained: vitamin A 175 IU, thiamine nitrate 700 mcg, vitamin B2 1.1 mg; vitamin B6 1.3 mg, nicotinamide 12 mg, vitamin C 60 mg, vitamin D3 100 IU, vitamin B12 1.5 mcg, vitamin E6 IU, biotin 50 mcg, vitamin B5 2.7 mg, folic acid 100 mcg, calcium hydrogen phosphate anhydrous 33.9 mg, ferrous fumarate 7.5 mg, magnesium oxide 8.32 mg, manganese sulphate 77 mcg, zinc oxide 1.25 mg, copper gluconate 178.6 mcg, and potassium iodide 118 mcg ^10^ LC-PUFA containing PLs supplement also contained phospholipids: 300 mg phosphatidylserine, 66 mg phosphatidylethanolamine, 48 mg phosphatidic acid, 24 mg lysophospholipids, 18 mg phosphatidylcholine, 12 mg phosphatidylinositol.

**Table 3:** Recalculation of measured blood fractions in included studies to Omega-3 Index equivalence.

				O3I Equivalence Before		O3I Equivalence After		O3I Equivalence Change		Significant Effect on Cognition?
	DHA, EPA Dose per Day	Blood Fraction	Formulae Used ^1^	Intervention	Placebo	Intervention	Placebo	Intervention	Placebo	
**Typically developing**										
Antypa [27]	250 mg DHA, 1740 mg EPA	Plasma FA	0.94 x + 1.17	3.46	3.18	6.28	3.36	2.82	0.18	Y
Baumgartner ^2^ [28]	240 mg DHA, 46 mg EPA	Total PP fraction of erythr membranes		3.20 ^a^ 3.17 ^b^	3.22 ^c^ 3.24 ^d^	6.27 ^a^ 6.33 ^b^	4.03 ^c^ 3.97 ^d^	3.07 3.16	0.81 0.73	N
Jackson ^3^ [33]	DHA rich group: 450 mg DHA, 90 mg EPA EPA rich group: 300 mg EPA, 200 mg DHA	Serum FA	0.94 x + 1.17	3.34 ^e^ 3.31 ^f^	3.16	4.21 ^e^ 4.24 ^f^	3.29	0.87 0.93	0.13	Y
Muthayya [41]	86 mg DHA	Erythr membranes in PP fraction	-	3.37 3.38	3.37 3.48	5.55 5.59	3.85 3.86	2.18 2.21	0.48 0.38	N
Hamazaki [32]	1500–1800 mg DHA, 200–240 mg EPA^4^	Total serum FA	0.94 x + 1.17	4.93	5.96	9.25	5.68	4.32	−0.28	N
Ryan [46]	400 mg DHA	Capillary WB	1.10 x + 0.65	2.08	2.08	4.83	2.19	2.75	0.11	N
Van der Wurff [47]	280 mg DHA, 520 mg EPA	Capillary WB	-	3.72	3.83	4.86	3.98	1.03	0.15	N
With disorder/disease										
Stevens [56]	480 mg DHA, 80 mg EPA	Eryth membranes	-	4.08	3.78	8.83	3.53	4.75	−0.25	Y
Widdenhorn-Muller [59]	120 mg DHA, 600 mg EPA	Eryth membranes	-	4.13	4.05	7.82	4.03	3.69	−0.02	Y

DHA = docosahexaenoic acid, EPA = eicosapentaenoic acid, erythr = erythrocytes, FA = fatty acids, n = no, O3I = Omega-3 Index, PP = phospholipids, y = yes, WB = whole blood. ^1^ For some blood fraction no conversion formulae is needed these are indicated with a -. ^2^ In the study of Baumgartner a 2 × 2 design was utilized the data presented here are for: placebo + DHA/EPA^a^, iron + DHA/EPA ^b^, iron+ placebo^c^, placebo + placebo^d^. ^3^ In the study of Jackson et al. two kinds of *n*-3 LCPUFA supplementation were utilized, the data presented here are for: DHA-rich fish oil^e^, EPA-rich fish oil^f^ .^4^ Supplementation dose was dependent on body weight.

## Appendix A

**Table A1:** Search Strategies.

PUBMED Search Strategy	(((“fatty acid”[Title/Abstract]) OR “fatty acids”[Title/Abstract]) OR “omega-3”[Title/Abstract]) OR “omega-6”[Title/Abstract]) OR pufa[Title/Abstract]) OR lcpufa[Title/Abstract]) OR mufa[Title/Abstract]) OR fish oil*[Title/Abstract]))) AND ((child [Title/Abstract] OR children [Title/Abstract] OR childhood [Title/Abstract] OR kid [Title/Abstract] or kids [Title/Abstract] OR girl* [Title/Abstract] OR boy* [Title/Abstract] OR teenage* [Title/Abstract] OR youth* [Title/Abstract] OR youngster* [Title/Abstract] OR preschool* [Title/Abstract] OR pre-school* [Title/Abstract] OR kindergarten* [Title/Abstract] OR “elementary school” [Title/Abstract] OR elementary-school [Title/Abstract] OR juvenile* [Title/Abstract] OR minors [Title/Abstract] OR minor [Title/Abstract] OR pediatric* [Title/Abstract] OR paediatric* [Title/Abstract] OR schoolchild*[Title/Abstract] OR toddler*[Title/Abstract] OR teen [Title/Abstract] OR teens [Title/Abstract] OR child, preschool [MeSH terms] OR child [MeSH terms])) AND (cognit*)Searched 03–07-2019–421 hits	
Web of Science Search Strategy	#1	(TS = (“fatty acid” OR “fatty acids” OR “omega-3” OR “omega-6” or pufa OR lcpufa OR mufa OR “fish oil*”)) Indexes = SCI-EXPANDED, SSCI, A&HCI, ESCI Timespan = All years
	#2	(TS = (child OR children OR childhood OR kid OR kids OR girl* OR boy* OR teenage* OR youth* OR youngster* OR preschool* OR pre-school* OR kindergarten* OR “elementary school” OR elementary-school OR juvenile* OR minors OR minor OR childhood OR pediatric* OR paediatric* OR schoolchild OR toddler* OR teen OR teens)) Indexes = SCI-EXPANDED, SSCI, A&HCI, ESCI Timespan = All years
	#3	(TS = (cognit*)) Indexes = SCI-EXPANDED, SSCI, A&HCI, ESCI Timespan = All years #3 AND #2 AND #1 Indexes = SCI-EXPANDED, SSCI, A&HCI, ESCI Timespan = All years Searched 03-07-2019-469 hits
